# ADPRH is a prognosis-related biomarker and correlates with immune infiltrates in low grade glioma

**DOI:** 10.7150/jca.51643

**Published:** 2021-03-15

**Authors:** Chunyu Zhang, Long Wang, Haitao Liu, Gang Deng, Pengfei Xu, Yinqiu Tan, Yang Xu, Baohui Liu, Qianxue Chen, Daofeng Tian

**Affiliations:** 1 Department of Neurosurgery, Renmin Hospital of Wuhan University, Wuhan, 430060, Hubei Province, P.R.C.; 2 Department of Cardiothoracic Surgery, The First Affiliated Hospital of Jiaxing University, Jiaxing, 314001, Zhejiang Province, P.R.C.; 3 Sun Yat-sen University, The Seventh Affiliated Hospital, Shenzhen, 518000, Guangdong Province, P.R.C.

**Keywords:** low grade glioma, immune infiltration, biomarker, TCGA, CGGA

## Abstract

**Background:** ADPRH is a modulator of CD8+ T cell functions, and dysregulation of ADPRH has been identified to involve in carcinogenesis of cancers. However, the association of ADPRH with low grade glioma (LGG) remains unclear.

**Methods:** The expression of ADPRH in LGG was first analyzed in GLIOVIS and GEPIA databases and then validated by real-time PCR (rt-PCR), immunochemistry and human protein atlas (HPA). Univariate and multivariate Cox analysis and Kaplan-Meier plots were designed to assess the prognostic value of ADPRH in LGG. The correlation of ADPRH and immune infiltration was evaluated by data in TIMER and ESTIMATE databases. Gene set enrichment analysis was conducted to detect biological processes associated with ADPRH.

**Results:** ADPRH was significantly upregulated in LGG in comparison to non-tumor brain samples in transcriptomic and proteomic levels. The high ADPRH expression indicated unfavorable overall survival (OS) and progression-free survival (PFS) in patients with LGG using Kaplan-Meier plots. And multivariate Cox analysis demonstrated the expression level of ADPRH was an independent prognosis-predicting index for OS and PFS of LGG patients in all cohorts separately. Gene Set Enrichment Analysis (GSEA) indicated that high expression of ADPRH was involved in the upregulation of P53 signaling pathway, KRAS signaling pathway, IL6/JAK-STAT3 signaling and TNF-beta signaling pathways. By TIMER and ESTIMATE databases, we identified ADPRH expression had strong correlation with tumor immune infiltrating cells (TIICs).

**Conclusions:** In summary, our findings demonstrated that ADPRH might be a potential prognostic biomarker and correlated with TIICs in LGG.

## Introduction

Low grade glioma (LGG) is a common tumor in central nervous system (CNS), comprising WHO grades II and III gliomas 1. Although molecular characteristics including isocitrate dehydrogenase 1 and 2 genes (IDH1/2), the codeletion status of chromosome arms 1p and 19q have significantly tell apart different diffuse glioma categories, however, LGG has great heterogeneity, hindering the improvement of patient outcomes 2. Despite the numerous advancements in adjuvant therapy and improvement in the surgical operation over the past decades, remarkable improvement in the clinical outcomes of LGG patients has not been made 3. With the exception of traditional treatments, there are growing interests in the emerging immunological approaches 4. One cause of the increasing attention to immune therapy is that patients developing resistance to conventional therapeutic strategies in all probability receive benefits from the novel methods 5. Neoplasms are distinguished by the cumulation of distinct genetic variations that lead to the expression of tumor antigens to activate anti-tumor immunological reaction 6, 7. However, there are complicated mechanisms in tumor cells of immune tolerance in cancers 8, 9, a major limitation of clinical application of anti-tumor immunotherapies. Meanwhile, functions of tumor infiltrating lymphocytes (TILs) could be blocked by varieties of immune cells. The upregulated T-regs impair function of cytotoxic T-cells to promote tumor cells to escape the control of immune system, for instance 10. High infiltration level of M2 macrophages have been considered to involve in the production and induction of angiogenic factors such as vascular endothelial growth factor (VEGF) and inhibitors of immune system such as transforming growth factor beta 1 (TGFB1) to facilitate cancer cell proliferation and invasion 11.

Recent years, accumulating research enable the researchers to gain insights into the sophisticated immune modulation and to explore effective checkpoints to regulate anti-tumor immune response, including the development of antibody drugs targeting the immunosuppressive molecules PD1 and PD-L1 in the medical treatment of renal cell cancer, prostate cancer and so on, which have demonstrated great effectiveness in clinical practice 12-15. Although the progression-free survival (PFS) and overall survival (OS) have improved significantly after receiving immunotherapy, a large proportion of patients often interrupted the treatment due to the side-effects 16. Therefore, molecular biomarkers with high reliability remain to be developed that can identify patients who benefit from immunotherapy and predict the prognosis of cancer patients.

Protein ADP-ribosylation, an invertible process in post-translational modification, is modulated by varieties of ADP-ribosylation transferase enzymes, which catalyze the ADP-ribosylation reactions using β-NAD+ derived ADP-ribose units and have been recognized to be implicated in DNA damage response, immunity, etc. 17, 18. Acting as the only enzyme in charge of the hydrolysis of ADP-ribosylated arginine in the ADP-ribosylation cycle, the enzyme encoded by ADPRH participates in the process of removing mono-ADP-ribose from arginine residues of proteins in the ADP-ribosylation cycle 18. Meanwhile, recent research demonstrates ADPRH expression is upregulated in regulatory T lymphocytes (Treg) in colorectal cancer (CRC) and non-small cell lung cancer (NSCLC) by RNA sequencing and single-cell PCR and tend to be associated with lymphocytes activation 19. However, the biological roles acted by ADPRH in LGG oncology and immunology are still ill-defined.

Here, we analyzed expression level of ADPRH in LGG samples in transcriptomics and proteomics, calculated the prognostic value of ADPRH expression, and identified the correlative degree of ADPRH expression with tumor immune microenvironment (TIM) in LGG patients.

## Materials and methods

### Data preparation

Total 1526 observations were included in our research. For total 1149 samples in TCGA, CGGA, GSE107850 cohorts, the inclusion criteria were (a) complete available follow-up information; (b) no comorbidity; (c) tumor grade was identified according to 2016 WHO classification. The RNA-seq data of 956 LGG observations included 404 LGG samples from the TCGA database (https://portal.gdc.cancer.gov) and 552 from the CGGA cohort (http://www.cgga.org.cn). Microarray data of 193 LGG samples in GSE107850 cohort were obtained from GEO database (https://www.ncbi.nlm.nih.gov/geo). Demographics of the above patients were described in Table S1. Microarray data of Rembrant cohort including 225 LGG and 28 non-tumor samples and Gravendeel dataset including 117 LGG and 7 non-tumor observations were acquired from GLIOVIS database (http://GLIOVIS.bioinfo.cnio.es/) for differential expression analysis.

### Human tissue samples

17 non-tumor brain tissues were obtained from patients with severe traumatic brain injury during surgery. 30 tumor specimens were collected during operation and pathologically examined as LGG at Renmin Hospital of Wuhan University, according to the 2016 WHO classification. Meanwhile, the enrolled patients with LGG were not treated with chemo-therapy or radio-therapy before surgery. Demographics of the patients in our cohort were described in Table S2. Written informed consent was obtained from all participants and this research by the Institutional Ethics Committee of the Faculty of Medicine at Renmin Hospital of Wuhan University [approval number: 2012LKSZ (010) H].

### Differential expression analysis

ADPRH expression in LGG and normal tissues was analyzed using the GEPIA (http://gepia.cancer-pku.cn) and GLIOVIS databases and validated by our own cohort using real‑time PCR (rt-PCR). The representative proteins immunohistochemistry (IHC) staining images of ADPRH were detected in LGG and normal tissues from human protein atlas (HPA) (https://www.proteinatlas.org/).

### Statistical analysis

The association between the expression level of ADPRH in LGG samples and OS and PFS were first assessed using Kaplan-Meier (K-M) plot. Univariate and multivariate Cox analysis were performed to further evaluate whether expression level of ADPRH was a statistically significant factor associated with OS and PFS, even adjusted by clinical variables (including age at diagnosis, gender, etc.). All the analyses were conducted on R project (version 3.6.3), Student's t-test were employed to compare ADPRH expression among distinct groups, p-value less than 0.05 was set up as the threshold.

### Gene set enrichment analysis

Gene set enrichment analyses (GSEA) was conducted to determine the biological pathway differences between the two groups, grouped by the median expression of ADPRH. h.all.v7.1.symbols.gmt was used as a reference get set. Pathways with p < 0.05 and FDR < 0.05 were considered to be remarkably changed.

### Immune infiltration analysis

Estimation of Stromal and Immune cells in Malignant Tumor tissues using Expression data (ESTIMATE) is a tool to calculate the degree of immune cell infiltration in the TIM in tissues. The calculated immune scores of TCGA LGG cohort were downloaded from ESTIMATE database. LGG patients were split into two groups, in accordance with the median immune score. TIMER database includes the abundance of TIICs by devolution of gene expression profiles in TCGA database. We conducted the correlation analysis between ADPRH expression and the abundance of TIICs, including CD4+ T cells, CD8+ T cells, B cells, neutrophils, dendritic cells and macrophages. The marker genes of immune infiltrating cells were acquired from previous studies 20-22. The correlation between the abundance of immune cells and markers and ADPRH was examined by Spearman test.

### Real‑Time PCR (RT‑PCR) analysis

Total RNA sequence was extracted using PrimeScriptTM RT Reagent Kit with a gDNA Eraser (Takara Bio Inc, Japan) according to manufacturer protocol and transcribed into cDNA. RT‑qPCR was carried out by SYBR Premix Ex Taq (Takara Bio Inc, Japan). The following primer sets were used for rt-qPCR: ADPRH-F 5'-GCGTTCCAGTCCAGGCTAC-3'; ADPRH - R 5'-GAGGGTGTCCAAGGTCTAGTT -3'; β-actin-F 5'-ATGGATGACGATATCGCTGCGC-3'; β-actin-R 5'-GCAGCACAGGGTGCTCCTCA-3'. β-actin was used for normalization.

### Immunohistochemistry

In this step, the sections were deparaffinized, hydrated and subjected to antigen retrieval in 10 mM sodium citrate (pH = 6.0). Endogenous peroxidase was inactivated in 3% H2O2 for 30 min. The sections were incubated in primary antibodies (Proteintech, China) overnight, followed by secondary antibody (Servicebio, China). Signals were detected using DAB staining (Servicebio, China). We acquired images by the Olympus BX51 microscope (Olympus). Two pathologists examined and scored the slides. IHC scores were described to estimate immunoreactivity: 0 for background staining, 1 for faint staining, 2 for moderate staining, and 3 for strong staining. Scores 0-1 were regarded low expression, and scores 2-3 high expression.

## Results

### Differential expression analysis of ADPRH in LGG patients

**Figure 1:**
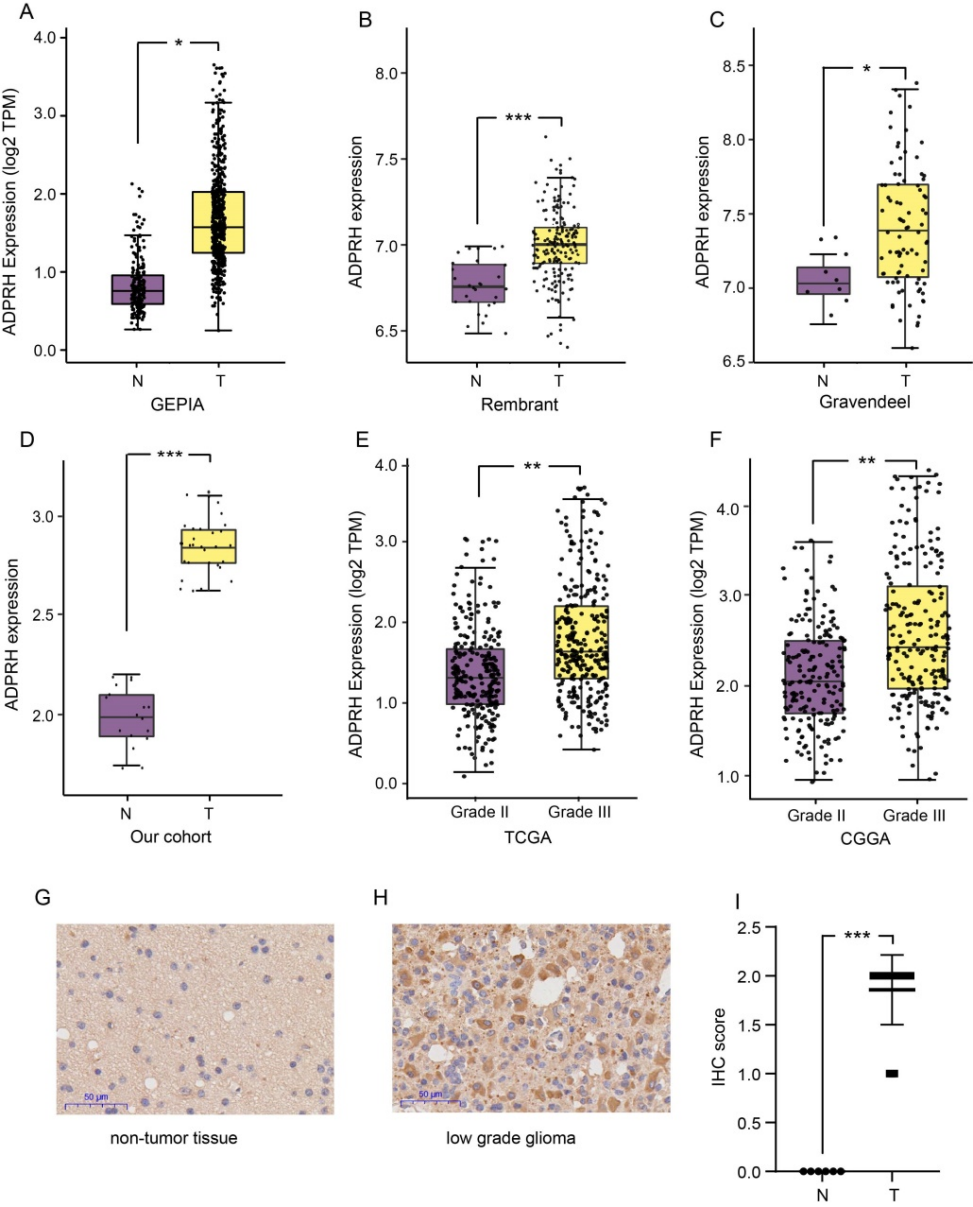
Expression analysis of ADPRH. Differential expression analysis of ADPRH in GEPIA database (A), Rembrant (B), and Gravendeel (C) cohorts from GLIOVIS database and validated by real time PCR (rt-PCR). ADPRH expression was normalized to β-actin using the 2-ΔΔCt method (D). ADPRH expression significantly correlates with WHO grade of glioma (E-F). Immunochemistry staining of ADPRH in LGG and non-tumor brain tissues (G-H), magnification, 200×. IHC score of ADPRH in clinical tissues (I), non-tumor tissue, n = 6; low-grade glioma, n = 28. T, tumor; N, non-tumor.

The expression level of ADPRH in LGG and non-tumor samples were first analyzed using the GEPIA database, revealing that the ADPRH was overexpressed in LGG samples in comparison to the normal brain tissues (Figure 1A). Based on GLIOVIS database, consistent results were obtained in the two datasets (Figure 1B-C). Our cohort containing 18 LGG samples and 10 non-tumor brain samples further validated ADPRH was expression-upregulated in LGG tissues (Figure 1D). To examine the association between ADPRH expression and WHO grading of LGG, we used the data from TCGA and CGGA cohorts. Figure 1E and 1F demonstrated expression of the key gene was positively correlated with tumor grade. IHC indicated that ADPRH was remarkably overexpressed in LGG sample in proteomic level, in comparison with the expression of ADPRH in normal brain tissue (Figure 1G-I). Representative IHC staining images from HPA further validated the findings (Figure S1).

### Analysis of ADPRH expression with survival

Using the TCGA, CGGA, and GSE107850 datasets, we launched an investigation into the prognosis-predicting potentiality of ADPRH for LGG patients. Patients were firstly split into two groups, in accordance with the median expression of the candidate gene in each dataset.

**Figure 2:**
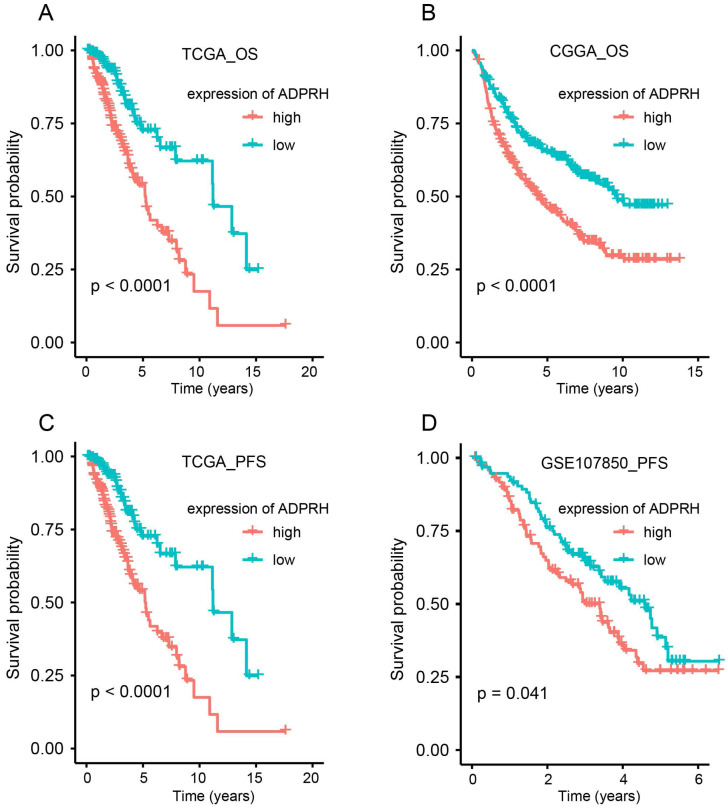
Kaplan-Meier (K-M) plots of the associations of ADPRH expression with OS in TCGA (A) and CGGA (B) cohorts and with PFS in TCGA (C) and GSE107850 datasets (D). OS, overall survival; PFS, progression-free survival.

K-M plot indicted that high expression of ADPRH was remarkably related to poor OS in TCGA (p < 0.001) and CGGA (p < 0.001) cohorts and PFS (p < 0.001) in TCGA and GSE107850 (p = 0.041) datasets (Figure 2A-D). Subsequently, univariate Cox regression analysis of patients in CGGA and TCGA demonstrated grade, age, IDH status, and ADPRH expression level were significantly relevant to OS (Table S3). And univariate Cox regression analysis of patients in GSE107850 and TCGA cohorts revealed ADPRH expression level was also associated with PFS of LGG patients (Table S4). In multivariate Cox regression analysis, ADPRH expression was significantly related to poor OS (TCGA: HR = 2.837, p < 0.001; CGGA: HR = 1.633, p < 0.001) (Table 1) and poor PFS (TCGA: HR = 1.701, p = 0.004; GSE107850: HR = 1.697, p = 0.018) (Table 2) in LGG patients. Therefore, the expression level of ADPRH was a factor, which could strongly and independently predict OS and PFS of patients with LGG.

### Significant pathways obtained by GSEA

**Figure 3:**
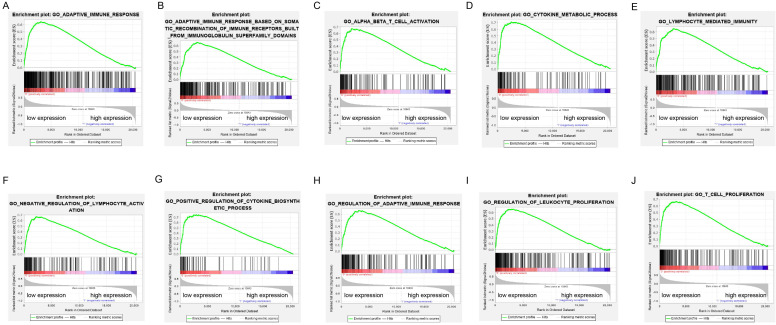
Enrichment plots of gene set enrichment analysis (GSEA) using the TCGA gene expression profiles of LGG.

GSEA was performed to verify the associated biological processes and signaling pathways using ADPRH on the basis of expression level for classification in TCGA cohort. Over expression of ADPRH was associated with several immune- and cancer-related processes and pathways including IL6/JAK-STAT3 signaling pathway, mTORC1 signaling pathway, P53 signaling pathway, KRAS signaling pathway, angiogenesis, epithelial-mesenchymal transition (EMT) and TNF-beta signaling pathway, demonstrating ADPRH in all possibility involved in the facilitation of cancer proliferation and immunosuppression (Figure 3A-J).

### Correlation analysis between ADPRH expression and immune infiltration

**Figure 4:**
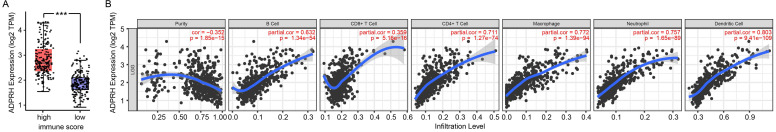
Correlation of ADPRH expression with immune infiltration level in LGG. Comparison of ADPRH expression between the high and low immune score groups of LGG (A). ADPRH is significantly correlated to tumor purity and immune infiltration cells in LGG tissues (B).

**Figure 5:**
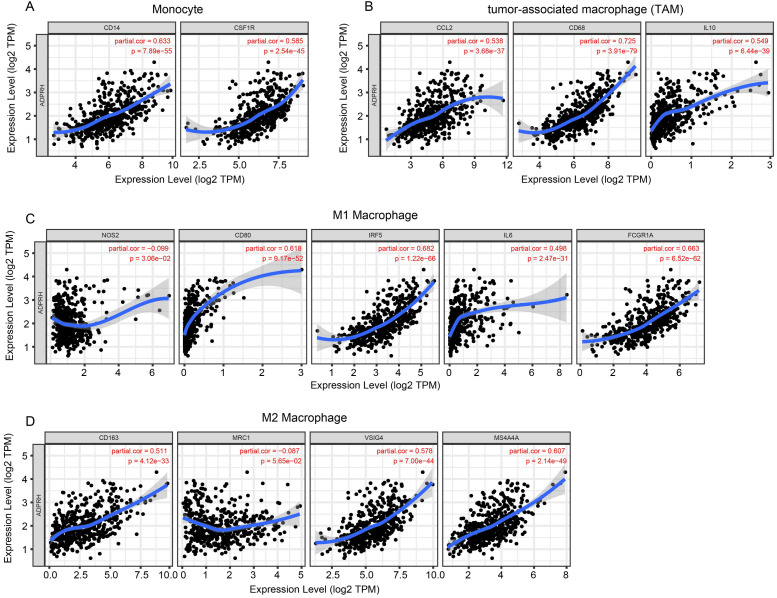
Correlation of ADPRH expression with marker genes of monocyte (A), tumor-associated macrophage (TAM) (B), M1 (C) and M2 macrophages (D) in LGG.

The infiltration levels of TIICs in tumors act a vital part in the tumor environment and affects prognosis of cancer patients 23. Here, the immune scores of patients were downloaded in TCGA LGG cohort from the ESTIMATE database to evaluate infiltration level of immune cells in tissues. And we noticed that patients with higher immune scores had higher ADPRH expression (p < 0.001), in comparison with the low immune score group (Figure 4A). Meanwhile, we analyzed the correlation between ADPRH expression and 6 types of TIICs using Spearman test in TIMER database. Figure 4B showed that ADPRH expression level had a significantly positive correlation with infiltrating levels of B cells (cor = 0.632, p = 1.34e-54), CD8+ T cells (cor = 0.359, p = 5.16e-16), CD4+ T cells (cor = 0.711, p = 1.27e-74), macrophages (cor = 0.772, p = 1.39e-94), neutrophils (cor = 0.757, p = 1.65e-89), and dendritic cells (cor = 0.803, p = 9.41e-109). After the correlation adjustment by purity, we discovered that the ADPRH expression was correlated with most of the gene markers of immune cells (Table 3), in particular, strongly and positively with the markers of monocytes, tumor-associated macrophages (TAMs), M1 and M2 macrophages (Figure 5).

In addition, there were high correlative degrees between ADPRH expression and markers of T cell exhaustion. TIM-3, a critical modulator of T cell exhaustion, strongly correlated with ADPRH expression (cor = 0.777, p < 0.001), demonstrating that ADPRH acted a pivotal part in TIM-3 mediating T cell exhaustion (Table 3).

## Discussion

The antibodies targeting crucial molecular in the immune system has already revealed remarkable progress in the clinical practice of cancers 24-26. In previous studies, immune-related markers have been explored and validated as independent prognostic factors in several tumors 27, 28. However, the research of prognostic biomarker related to tumor immune infiltration in the field of LGG has not been well conducted until now. We found that ADPRH was expression-upregulated in LGG samples, and correlated with poorer OS and PFS. Meanwhile, there was strong correlation between ADPRH and immune infiltration in LGG tissues. These findings demonstrated there was great potential for ADPRH acting as a biomarker for prognosis and a target for immunotherapy.

ADPRH specifically serves as an arginine mono-ADP-ribosylhydrolase by mediation of removing mono-ADP-ribose adhered to arginine residues on proteins. Recent research shows that ADPRH plays a key role in the formation of lung adenocarcinoma and lymphoma 29 and involves in regulation of lymphocytes activation in lung and colorectal cancers 19. However, the expression pattern and biological role of ADPRH in LGG has not been studied until now. In this study, ADPRH over-expression was identified in LGG tissues in transcriptomics and proteomics level in comparison with normal brain tissues. In addition, our results indicated ADPRH expression was significantly associated with an increase in the grade of malignancy in LGG. K-M plot demonstrated patients with higher expression level of ADPRH had reduction in OS and PFS compared to those with low expression in LGG. To further explore the prognostic significance of ADPRH, univariate and multivariate Cox analysis were conducted. We identified and validated the expression level of ADPRH was a potent and independent prognostic indictor for observations with LGG. When ADPRH over-expressed in LGG, we found some inflammation-associated and oncogenic pathways upregulated such as P53 signaling, KRAS signaling, IL6/JAK-STAT3, and TNF-beta signaling pathways. In the tumor microenvironment, hyperactivation of IL-6/JAK/STAT3 signaling pathway acts as a powerful suppressor of anti-tumor immune response and as a promotor of tumor progression, leading to a poor prognosis of cancer patients 30. In the regulation axis, STAT3 could facilitate the expression of IL-6, and also induces the expression of angiogenic and immunosuppressive factors including VEGF and TGFB1. Meanwhile, IL-6 also induces the expansion of pro-inflammatory and angiogenesis-promoting factors, including C-C motif chemokine 2 (CCL2), and VEGF. TGFB1 has been recognized as the most predominant immune-suppressing molecular by inducing Treg cells infiltration into tumor microenvironment and by inhibiting the generation, differentiation and function of effector T cells 10. Meanwhile, TGFB1 disrupts the antigen presentation ability of dendritic cells by inhibiting the expression of MHC-II genes 31, 32.

Recent years have witnessed remarkable development and advancements in the research of tumor immunotherapy and the role of the immune response in the tumorigenesis and progression has become the focus studied and front field, and demonstrated the excellent development prospect. Recent research has demonstrated that immune cell infiltration exerts a profound impact on prognosis in neoplasms 33, 34. When analyzing the correlation between ADPRH expression with immune infiltration levels in LGG, our results demonstrated that there were strong positive relationships between ADPRH expression level and infiltration level of Monocytes, TAMs, B cells, CD8+ T cells, CD4+ T cells, neutrophils and DCs, revealing that ADPRH may possibly involve in tumor immune infiltration. Meanwhile, markers of monocyte, M1 and M2 macrophages and T cell exhaustion such as CD68, CD86, IRF5, and TIM-3 strongly correlated with the expression of ADPRH. These results demonstrated the possible modulating function of ADPRH in polarization of TAMs and induction of T cell exhaustion. Recent studies reveal TAMs have negative impacts on prognosis of cancer patients 33, 34. Macrophages are recruited to the hypoxic cores within tumor and induce factors associated angiogenesis and proteases related to tumor invasion and progression, such as matrix metalloproteinase 9 (MMP-9) 35. Meanwhile, M2 macrophages also play a suppressing role in anti-tumor activity by producing angiogenic and anti-inflammatory factors 35, 36. In glioma, M2 macrophages promote tumor cell proliferation by activating STAT3, resulting in prognosis of patients with glioma 37. Here, ADPRH expression had significantly correlative levels with the abundance of M2 macrophages and markers of M2 macrophages, which was a possible mechanism for ADPRH leading to poor prognosis of LGG patients.

There are several limitations in this research. First, expression level of ADPRH was validated to be associated with PFS and OS of LGG patients, however, the prognostic value of ADPRH in clinical practice remained to be studied further. Second, the underlying mechanisms of ADPRH with TIICs in LGG remain unclear, while GSEA results provided several clues. Further investigations are needed into the detailed mechanisms of the correlation between ADPRH and immune cells infiltration in LGG tissues.

## Conclusions

In summary, our comprehensive analysis revealed that ADPRH was upregulated in LGG, and high expression of ADPRH was related to poor prognosis for LGG patients. Additionally, ADPRH may modulate anti-tumor immune response and promote the proliferation and progression of LGG. Therefore, we report that ADPRH is a possible prognostic biomarker and an immune-associated therapeutic target for LGG.

## Supplementary Material

Supplementary figures and tables.

## Figures and Tables

**Table 1:** Multivariate Cox regression analysis of OS of LGG in TCGA and CGGA cohorts

Covariates	TCGA cohort (n=404)			CGGA cohort (n=552)		
	HR	95% CI	P	HR	95% CI	P
Grade (ref. WHO II)	1.903	1.189-3.046	**0.007**	2.916	2.237-3.800	**<0.001**
Gender (ref. Female)	1.166	0.776-1.753	0.460	0.955	0.752-1.214	0.709
Age (continuous, years)	1.053	1.034-1.072	**<0.001**	1.007	0.996-1.018	0.244
IDH status (ref. Mutant)	2.636	1.503-4.624	**0.001**	1.587	1.190-2.115	**0.002**
ADPRH (continuous)	2.837	1.759-4.575	**<0.001**	1.633	1.359-1.955	**<0.001**

OS, overall survival; HR, hazard ratio; CI, confidence interval.

**Table 2:** Multivariate Cox regression analysis of PFS of LGG in TCGA and GSE107850 cohorts

Covariates	TCGA cohort (n=404)			GSE107850 cohort (n=193)		
	HR	95% CI	P	HR	95% CI	P
Grade (ref. WHO II)	1.071	0.759-1.512	0.697	-	-	-
Gender (ref. Female)	0.901	0.660-1.230	0.511	1.170	0.775-1.766	0.455
Age (continuous, years)	1.017	1.004-1.030	**0.009**	1.009	0.960-1.039	**0.026**
IDH status (ref. Mutant)	3.720	2.394-5.779	**<0.001**	2.412	1.481-3.931	**<0.001**
ADPRH (continuous)	1.701	1.191-2.430	**0.004**	1.697	1.096-2.626	**0.018**

PFS, progression-free survival; HR, hazard ratio; CI, confidence interval.

**Table 3:** Correlation analysis between ADPRH expression and related markers of immune cells using data in TIMER database

Description	Gene markers	LGG			
		None		Purity	
		Cor	P	Cor	P
CD8+ T cell	CD8A	0.351	***	0.259	***
	CD8B	0.376	***	0.305	***
	CD45	0.788	***	0.759	***
T cell (general)	CD3D	0.562	***	0.525	***
	CD3E	0.63	***	0.607	***
	CD2	0.63	***	0.617	***
B cell	CD19	0.462	***	0.431	***
	CD79A	0.324	***	0.331	***
	CD27	0.182	***	0.205	***
	CD20	0.306	***	0.279	***
Monocyte	CD14	0.652	***	0.633	***
	CD115 (CSF1R)	0.644	***	0.585	***
TAM	CCL2	0.563	***	0.538	***
	CD68	0.75	***	0.725	***
	IL10	0.579	***	0.549	***
M1 Macrophage	INOS (NOS2)	-0.071	ns	-0.099	*
	CD80	0.599	***	0.618	***
	IRF5	0.726	***	0.682	***
	IL6	0.523	***	0.498	***
	CD64 (FCGR1A)	0.704	***	0.663	***
M2 Macrophage	CD163	0.496	***	0.511	***
	CD206	-0.041	ns	-0.087	ns
	VSIG4	0.617	***	0.578	***
	MS4A4A	0.604	***	0.607	***
Neutrophils	CD66b (CEACAM8)	-0.016	ns	-0.014	ns
	CD11b (ITGAM)	0.704	***	0.655	***
	CD15	0.537	***	0.493	***
Natural killer cell	KIR2DL1	0.066	ns	0.088	ns
	KIR2DL3	0.224	***	0.216	***
	KIR3DL1	0.055	ns	0.061	ns
	KIR3DL2	0.239	***	0.233	***
	CD56	-0.408	***	-0.343	***
	CD335 (NKp46)	0.142	***	0.178	***
Dendritic cell	BDCA-1 (CD1C)	0.398	***	0.399	***
	BDCA-3 (CD141)	0.382	***	0.397	***
	BDCA-4 (NRP1)	0.309	***	0.379	***
	CD123	0.259	***	0.236	***
	CD11c (ITGAX)	0.676	***	0.627	***
Th1	T-bet (TBX21)	0.367	***	0.397	***
	STAT4	-0.041	ns	-0.12	*
	STAT1	0.503	***	0.512	***
Th2	GATA3	0.475	***	0.443	***
	STAT6	0.613	***	0.536	***
	IL13	-0.013	ns	-0.016	ns
Tfh	BCL6	0.036	ns	0.093	*
	IL21	0.109	*	0.107	*
Th17	STAT3	0.545	***	0.582	***
	IL17A	-0.034	ns	-0.025	ns
Treg	FOXP3	-0.093	*	-0.082	ns
	CD25	0.323	***	0.364	***
	CCR8	0.202	***	0.203	***
	STAT5B	-0.078	ns	0.033	ns
T cell exhaustion	PD-1 (PDCD1)	0.576	***	0.562	***
	CTLA4	0.21	***	0.25	***
	LAG3	0.807	***	0.777	***
	TIM-3 (HAVCR2)	0.354	***	0.382	***

LGG, low grade glioma; TAM, tumor-associated macrophage; Th, T helper cell; Tfh, Follicular helper T cell; Treg, regulatory T cell; Cor, R value of Spearman's correlation; None, correlation without adjustment; Purity, correlation adjusted by purity. ns, P > 0.05; *P < 0.05; **P < 0.01; ***P < 0.001.

## Abbreviations

LGG: low grade glioma
HPA: human protein atlas
rt-PCR: real-time PCR
OS: overall survival
PFS: progression-free survival
TIICs: tumor immune infiltrating cells
K-M: Kaplan-Meier
CNS: central nervous system
TILs: tumor infiltrating lymphocytes
VEGF: vascular endothelial growth factor
TGFB1: transforming growth factor beta 1
GSEA: gene set enrichment analyses
ESTIMATE: Estimation of Stromal and Immune cells in Malignant Tumor tissues using Expression data
EMT: epithelial-mesenchymal transition
