# Supplementary material for: ADPRH is a prognosis-related biomarker and correlates with immune infiltrates in low grade glioma
Source: J Cancer. 2021 Mar 15;12(10):2912–20. doi: 10.7150/jca.51643 (PMC8040889; doi:10.7150/jca.51643)
Supplement: Supplementary file 1 — Supplementary figures and tables. [file jcav12p2912s1.pdf]

## Supplementary materials

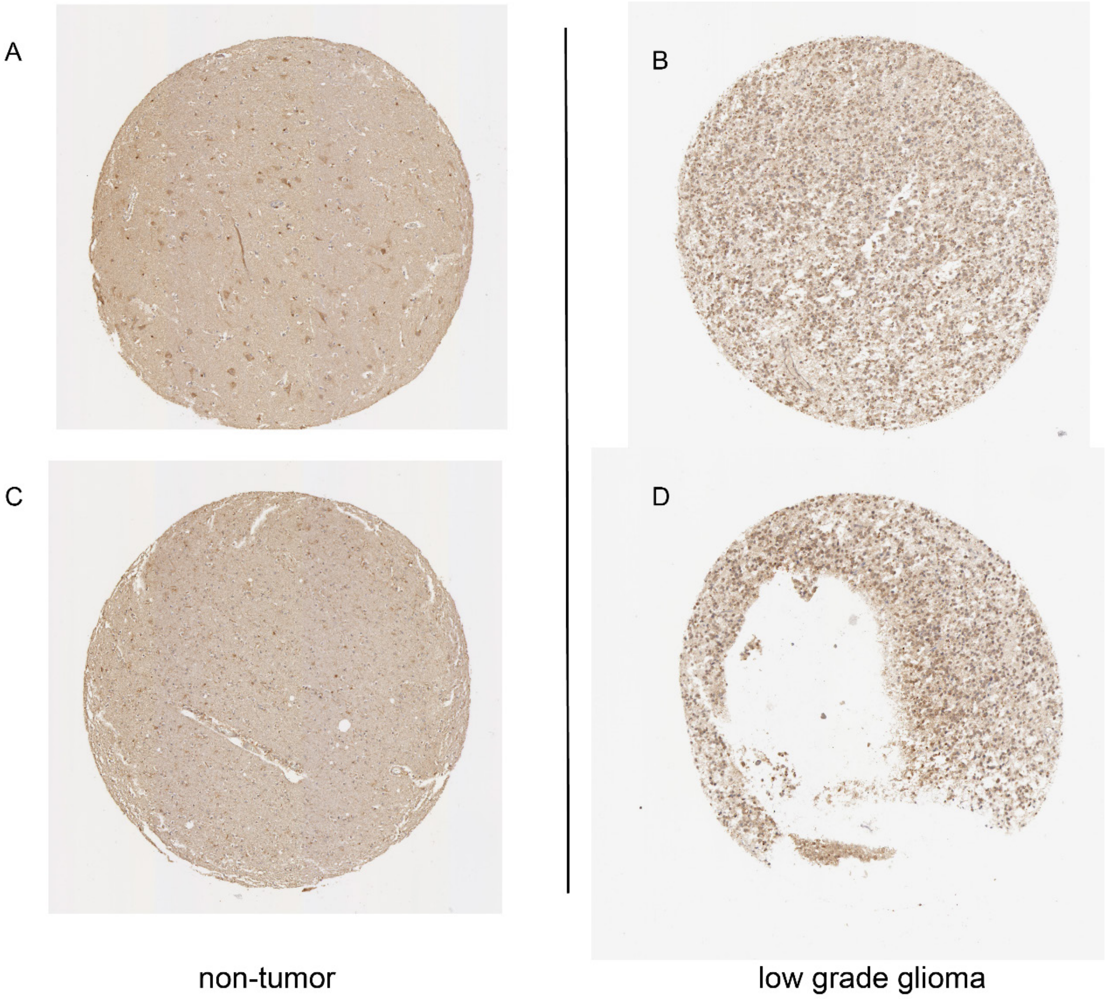


Figure S1. Representive immunochemistry staining images of ADPRH in non-tumor brain tissues (A, C), and low grade glioma (B, D).

Table S1. Demographics and characteristics of patients with low-grade glioma.

| **Variables** | **TCGA cohort (n=404)** | **CGGA cohort (n=552)** | **GSE107850 cohort (n=193)** |
|---|---|---|---|
| **Grade** | | | |
| WHO II | 192 | 255 | - |
| WHO III | 212 | 297 | - |
| **Gender** | | | |
| Male | 225 | 318 | 109 |
| Female | 179 | 234 | 84 |
| **Age** | | | |
| ≥41 | 210 | 265 | 117 |
| <41 | 194 | 287 | 76 |
| **IDH status** | | | |
| Wild-type | 75 | 137 | 29 |

| | | | |
|---|---|---|---|
| Mutant | 329 | 415 | 164 |

Table S2. Demographics of patients in our own cohort.

| ID | Gender | Age | location | Grade |
|---|---|---|---|---|
| 6002588 | F | 30 | Cerebellar Vermis | LGG |
| 1668128 | M | 44 | Right temporal lobe | LGG |
| 1657237 | M | 55 | Left frontal temporal lobe | LGG |
| 1647757 | M | 50 | Right temporal lobe | LGG |
| 1637666 | M | 56 | Spinal Cord | LGG |
| 1634247 | M | 40 | Right frontal lobe | LGG |
| 1625521 | M | 66 | Brain stem | LGG |
| 1617511 | M | 74 | Left parietal lobe | LGG |
| 1614653 | F | 34 | Left frontal temporal lobe | LGG |
| 1607245 | M | 30 | Left frontal lobe | LGG |
| 1603579 | F | 21 | Left frontal lobe | LGG |
| 1595565 | F | 46 | Left frontal lobe | LGG |
| 1590759 | M | 69 | Left frontal lobe | LGG |
| 1587231 | F | 65 | Right frontal lobe | LGG |
| 1586974 | F | 29 | Right frontal lobe | LGG |
| 1584150 | M | 31 | Left frontal lobe | LGG |
| 1581677 | M | 53 | Left parietal lobe | LGG |
| 1575864 | F | 43 | Right frontal lobe | LGG |
| 1572916 | F | 41 | Right frontal lobe | LGG |
| 1565440 | M | 42 | Right frontal lobe | LGG |
| 1555404 | F | 57 | Right temporal lobe | LGG |
| 1554108 | F | 59 | Right frontal lobe | LGG |
| 1547947 | M | 40 | Right temporal lobe | LGG |
| 1545992 | M | 55 | Left temporal lobe | LGG |
| 1537077 | M | 35 | Left parietal lobe | LGG |
| 1536012 | F | 47 | Left frontal lobe | LGG |
| 1533094 | M | 51 | Right temporal lobe | LGG |
| 1532352 | M | 32 | Left frontal temporal lobe | LGG |
| 1527837 | M | 30 | Right frontal temporal lobe | LGG |
| 1526810 | F | 46 | Right parietal lobe | LGG |
| 1597531 | F | 41 | Bilateral frontal lobes | NB |
| 1596317 | M | 27 | Left frontal lobe | NB |
| 1592526 | M | 47 | Left frontal lobe | NB |
| 1591578 | M | 42 | Left frontal lobe | NB |
| 1589735 | M | 44 | Bilateral frontal lobes | NB |
| 1587379 | M | 57 | Right Cerebellum | NB |
| 1582940 | F | 57 | Left frontal parietal lobe | NB |
| 1582844 | F | 49 | Left frontal parietal lobe | NB |
| 1573405 | M | 50 | Right frontal lobe | NB |
| 1573316 | F | 65 | Left temporal lobe | NB |

| | | | | |
|---|---|---|---|---|
| 1572794 | M | 27 | Left thalamus | NB |
| 1671777 | F | 25 | Left Cerebellum | NB |
| 1671687 | M | 33 | Right frontal lobe | NB |
| 1671265 | F | 36 | Left frontal lobe | NB |
| 1670530 | F | 16 | Right thalamus | NB |
| 1669226 | F | 39 | Intraventricle | NB |
| 1660396 | F | 20 | Right Cerebellum | NB |

Table S3. Univariate Cox regression analysis of OS in TCGA and CGGA cohorts.

| **Covariates** | **TCGA cohort (n=404)** | | | **CGGA cohort (n=552)** | | |
|---|---|---|---|---|---|---|
| | **HR** | **95% CI** | ***P*** | **HR** | **95% CI** | ***P*** |
| Grade (ref. WHO II) | 3.412 | 2.196-5.302 | <0.001 | 3.082 | 2.375-3.999 | <0.001 |
| Gender (ref. Female) | 1.068 | 0.721-1.583 | 0.742 | 0.907 | 0.715-1.151 | 0.422 |
| Age (continuous, years) | 1.057 | 1.040-1.074 | <0.001 | 1.015 | 1.002-1.027 | 0.018 |
| IDH status (ref. Mutant) | 6.835 | 4.535-10.302 | <0.001 | 2.300 | 1.787-2.960 | <0.001 |
| ADPRH (continuous) | 4.818 | 3.265-7.109 | <0.001 | 1.949 | 1.638-2.319 | <0.001 |

OS, overall survival; HR, hazard ratio; CI, confidence interval.

Table S4. Univariate Cox regression analysis of PFS in TCGA and GSE107850 cohorts.

| **Covariates** | **TCGA (n=404)** | | | **GSE107850 (n=193)** | | |
|---|---|---|---|---|---|---|
| | **HR** | **95% CI** | ***P*** | **HR** | **95% CI** | ***P*** |
| Grade (ref. WHO II) | 1.643 | 1.198-2.255 | 0.002 | - | - | - |
| Gender (ref. Female) | 0.846 | 0.621-1.152 | 0.288 | 1.36 | 0.911-2.031 | 0.133 |
| Age (continuous, years) | 1.027 | 1.015-1.039 | <0.001 | 0.973 | 0.955-0.991 | 0.004 |
| IDH status (ref. Mutant) | 5.866 | 4.153-8.285 | <0.001 | 2.675 | 1.664-4.300 | <0.001 |
| ADPRH (continuous) | 3.047 | 2.269-4.091 | <0.001 | 1.874 | 1.226-2.862 | 0.004 |

PFS, progression-free survival; HR, hazard ratio; CI, confidence interval.
